# Physical Activity in Pre-Ambulatory Children with Cerebral Palsy: An Exploratory Validation Study to Distinguish Active vs. Sedentary Time Using Wearable Sensors

**DOI:** 10.3390/s25041261

**Published:** 2025-02-19

**Authors:** Julie M. Orlando, Beth A. Smith, Jocelyn F. Hafer, Athylia Paremski, Matthew Amodeo, Michele A. Lobo, Laura A. Prosser

**Affiliations:** 1 Division of Rehabilitation Medicine, Children’s Hospital of Philadelphia, Philadelphia, PA 19104, USA; paremskia@chop.edu (A.P.); prosserl@chop.edu (L.A.P.); 2 Developmental Neuroscience and Neurogenetics Program, The Saban Research Institute, Children’s Hospital Los Angeles, Los Angeles, CA 90027, USA; bsmith@chla.usc.edu; 3 Division of Developmental-Behavioral Pediatrics, Children’s Hospital Los Angeles, Los Angeles, CA 90027, USA; 4 Department of Pediatrics, Keck School of Medicine, University of Southern California, Los Angeles, CA 90089, USA; 5 Kinesiology and Applied Physiology, University of Delaware, Newark, DE 19713, USA; jfhafer@udel.edu; 6 Department of Physical Medicine and Rehabilitation, Hospital of the University of Pennsylvania, Philadelphia, PA 19146, USA; matthew.amodeo@ochsner.org; 7 Department of Pediatric Neurosciences, Ochsner Health System, New Orleans, LA 70121, USA; 8 Physical Therapy Department, Biomechanics & Movement Science Program, University of Delaware, Newark, DE 19713, USA; malobo@udel.edu; 9 Department of Pediatrics, Perelman School of Medicine, University of Pennsylvania, Philadelphia, PA 19104, USA

**Keywords:** wearable sensors, activity cut-points, activity monitoring, motion sensors, accelerometry, actigraphy, sedentary, active, cerebral palsy

## Abstract

Wearable inertial sensor technology affords opportunities to record the physical activity of young children in their natural environments. The interpretation of these data, however, requires validation. The purpose of this study was to develop and establish the criterion validity of a method of quantifying active and sedentary physical activity using an inertial sensor for pre-ambulatory children with cerebral palsy. Ten participants were video recorded during 30 min physical therapy sessions that encouraged gross motor play activities, and the video recording was behaviorally coded to identify active and sedentary time. A receiver operating characteristic curve identified the optimal threshold to maximize true positive and minimize false positive active time for eight participants in the development dataset. The threshold was 0.417 m/s^2^ and was then validated with the remaining two participants; the percent of true positives and true negatives was 92.2 and 89.7%, respectively. We conclude that there is potential for raw sensor data to be used to quantify active and sedentary time in pre-ambulatory children with physical disability, and raw acceleration data may be more generalizable than the sensor-specific activity counts commonly reported in the literature.

## 1. Introduction

Time spent playing on the floor, or playing in an unconstrained environment, has important implications for child development [1,2,3,4]. For young children under three years of age, floor play time allows for self-initiated movement, exploratory behavior with objects, and face-to-face interactions with parents [1,5]. These early exploratory behaviors, including exploration of self and objects are predictive of motor, cognitive, and language development, and early exploratory behaviors are also related to more advanced play skills [3,4]. Young children tend to vary exploratory actions with toys and face-to-face interactions with their parents based on their position. Thus, encouraging unconstrained floor play time may provide opportunities for children to experience different positions and have varied, rich interactions with the objects and people within their environment [1,6]. Therefore, there is a need to develop novel ways to measure physical activity during floor play activities, which contribute to opportunities for developmental advancement for pre-walking children.

For children with cerebral palsy (CP) or who are at high risk for CP, floor play time may have even greater implications for development. Children with CP display more sedentary behaviors than typically developing peers [7]. Sedentary time appears to increase for children with CP over early childhood (i.e., 2–5 years of age) regardless of severity level, indicated by the gross motor function classification system (GMFCS) level [8], with level 1 indicating the lowest severity and level 5 indicating the highest severity. Additionally, 2-year-old children with CP (GMFCS levels 3, 4, and 5) were less likely to meet physical activity guidelines than their typically developing peers over a 3-day period [9,10]. Measuring activity during floor play time in pre-walking children will support our ability to identify children who have inadequate active time and implement interventions early in life.

While measuring activity for young children can provide valuable information, there are limitations with current measurement approaches. The gold standard for measuring and categorizing variable activities is the behavioral coding of video recordings; however, this method is time and resource intensive, and not always feasible in natural environments, such as home and childcare settings [11,12]. Some research groups have explored using machine learning to classify activities; however, there remain challenges with misclassifying activities, particularly during adult interactions [13,14,15,16,17,18]. Fully personalized machine learning classifiers were the most accurate in classifying activities for youth with CP, GMFCS level I–III, in a laboratory setting; however, they were found to have poor accuracy in a natural setting and require the presence of a researcher to identify activities during the training period [13]. The presence of the researcher may change participant behavior. For example, in a qualitative in-depth interview, mothers of 2-year-old children reported that their behavior and their child’s behaviors changed during parent–child observations, which they felt was related to the researcher’s presence [18,19]. The concept of “knowing you are being recorded” whether a researcher is present or not may also change behavior, and this limitation applies to multiple measurement systems, including wearable sensors.

Interpreting data recorded from wearable sensors is another significant area of challenge. The amount and type of activity (frequently reported as sedentary, light, or moderate-to-vigorous physical activity) is often reported in proprietary “activity counts”, which poses challenges for interpreting data recorded with different sensors and lacks methodological transparency for understanding the cut-points for activity level classifications [20,21,22,23,24,25]. For example, a higher activity count, suggesting a higher intensity of movement, can reflect a higher frequency of movement or larger acceleration of the movements produced and does not distinguish between those patterns. Further, the accurate measurement of activity requires validation in unique research cohorts, which has often not been performed when using existing “activity count” methods. In 2-month-old infants, for example, it is possible that many of their movements are “below threshold” and do not generate activity counts, thus providing an inaccurate output. Young infants can demonstrate a muscle strength gain to body growth/mass gain imbalance, which would likely result in slow acceleration movements [26]. As such, current acceleration thresholds for counting “activity counts” may be too high to adequately capture many of their movements [26]. In a scoping review examining physical activity measurements in children under 2 years old, Prioreschi and Micklesfield concluded that cut-points specific to this population are needed, and reporting “raw counts” should be prioritized to allow for comparisons across studies, particularly due to the variability in movements among infants and toddlers [27]. However, often “raw counts” have not been validated in the population being measured [28]. This study will use raw acceleration data from an inertial measurement unit to develop a threshold to determine active vs. sedentary time in pre-ambulatory children with CP.

There are a greater number of validation studies for ambulatory children. Hurter et al. established device-specific thresholds using raw accelerometry signals to classify sedentary and stationary activities in 9–10-year-old children [29]. Trost et al. validated cut-points for sedentary, light, and moderate–vigorous physical activity in ambulatory toddlers using triaxial accelerometers worn at the right hip [30,31]. As noted in a systematic review and meta-analysis conducted by Bruijns et al., these cut-points have been widely used in studies including toddlers [32]. In addition to the Trost et al. cut-points, often studies have applied cut-points that had not been validated for the toddler age range [31,32]. The validity of such results are questionable.

For young children, sensors have been validated to identify positions and to classify specific movement patterns. The Get Around Garment is a validated smart garment with an integrated triaxial accelerometer to classify infant positions in a natural environment [33]. Airaksinen et al. have also developed and validated a smart jumpsuit for infants incorporating multiple triaxial accelerometers and gyroscopes to classify specific movements and positions and gross motor skills using machine learning algorithms [34,35]. To identify specific movement patterns, infant leg movement quantity and kinematic characteristics have been validated, as well as the detection of infant arm movement bouts [36,37]. While validation studies have been growing with this young population, there remains a need to validate the use of sensors to quantify activity for young children. Ghazi et al. developed and validated physical activity thresholds for pre-walking infants based on the area under the acceleration–time curve and area under the jerk–time curve using synchronized behaviorally coded video recordings and ankle sensor recordings. However, the amount of data per participant was limited to 2–8 min [38]. Therefore, additional validation studies are needed with longer durations of data and samples across different age and motor skill ranges.

In order to better prescribe early activity interventions for pre-walking children with CP, objective methods to evaluate physical activity levels are needed. Using methods that can be applied to raw sensor data would make a physical activity monitoring tool accessible to and comparable across patient populations. Therefore, the purpose of this study was to develop and establish the criterion validity of a method of quantifying active and sedentary activity using triaxial accelerometer data from an inertial sensor worn on the lateral thigh for pre-walking children with cerebral palsy during floor play time.

## 2. Materials and Methods

### 2.1. Participants

Participants were pre-ambulatory children with CP recruited as part of two rehabilitation clinical trials: the first investigating the influence of two rehabilitation approaches on motor development in children with CP (ClinicalTrials.gov Identifier: NCT02340026) and the second characterizing the development of locomotor learning over the first 18 months of life in infants at high risk for CP (ClinicalTrials.gov Identifier: NCT04561232) [39,40]. See Table 1 for participant information. All procedures were approved by the Institutional Review Board at The Children’s Hospital of Philadelphia.

### 2.2. Data Collection

**Figure 1:**
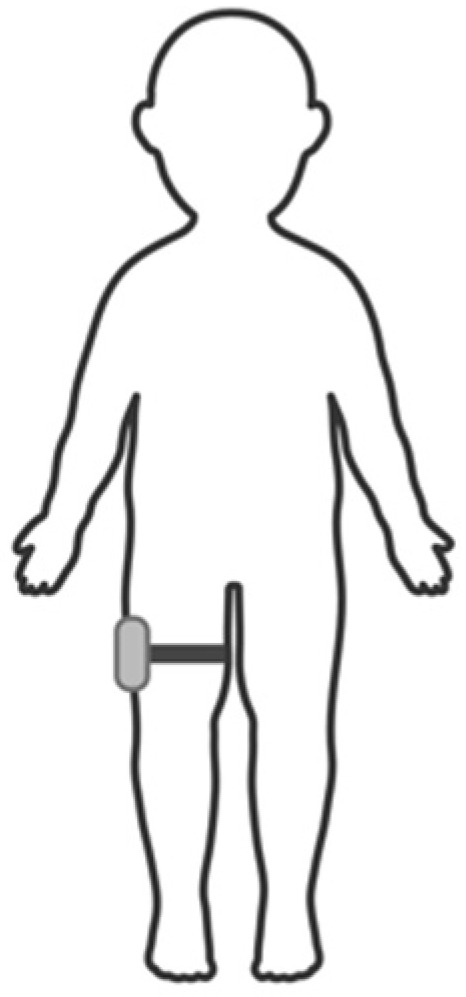
Schematic of sensor placement. Sensors were placed on the lateral aspect of the child’s dominant thigh. Created in BioRender. Orlando, J. (2024) https://BioRender.com/l01b058.

Participants were video recorded during a 30 min (mean: 30.6 min, range: 30.2–30.9 min) physical therapy session in an outpatient physical therapy environment between November of 2018 and June of 2023. The space included developmentally appropriate toys and surfaces to encourage movement and pulling-to-stand. The participants wore an inertial sensor (Opal sensor, APDM, Inc., Portland, OR, USA) on their dominant leg, placed at the lateral aspect of the child’s thigh and secured with a hook and loop fastening strap (Figure 1). The placement was chosen to capture a majority of gross motor movements. The sensor recorded data at 128 Hz. The sessions were video recorded at 30 Hz with a VIXIA HF R70 camcorder (Canon U.S.A Inc., Melville, NY, USA).

### 2.3. Data Processing

Behavioral coding was conducted using Datavyu, an open source software package that is designed for behavioral coding from video observations [41]. Our guidelines for coding active and sedentary time are described in Supplementary Material S1, including specific examples. Movement classified as “active” time included walking with a push-toy or with upper limb support, dynamic movement or transitions, and limb movement that included at least 30 degrees of trunk movement, while “sedentary” time was coded for static positions. Adult handling, or time when the participant was picked up or moved by an adult, was also identified and excluded during the threshold development, and then included as “sedentary” time during validation. Behavioral coding was conducted by researchers trained in video observation (AP, JO). Inter-rater frame by frame reliability was completed for 30 percent of the data and calculated as [the number of agreements/ (the number of disagreements + agreements)] × 100 within a tolerance of 500 milliseconds using Datavyu software (version 1.3.8).

The start time and stop times of the session recorded by the video and sensor data were synchronized. When possible, a button press on the sensor was used to synchronize the video and sensor data. In one case, the button press was not possible due to the version of the sensor, and instead, the video and sensor data were synchronized using a windmill action (i.e., moving the sensors in a large circle for 5 cycles) performed by the researcher prior to donning and again after removing the sensor from the child. These movements were easily identified in a graph of the raw (unfiltered) resultant acceleration magnitude data.

**Figure 2:**
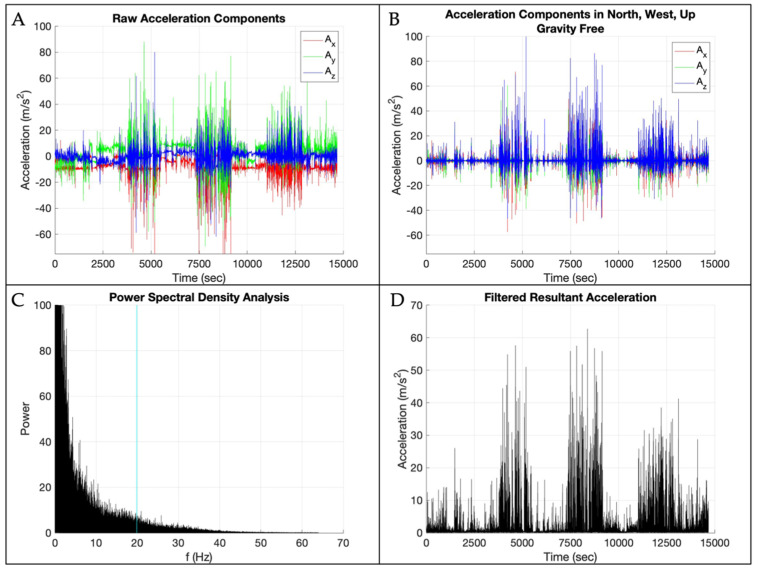
Data processing steps: (**A**) raw acceleration components with the x-axis in red, y-axis in green, and z-axis in blue; (**B**) raw acceleration transformed into the world frame of reference with gravity removed with the x-axis in red, y-axis in green, and z-axis in blue; (**C**) power spectral density results with the vertical blue line at 95% of the data; (**D**) filtered resultant acceleration magnitude.

Raw triaxial accelerometer data were visually inspected in Motion Studio (APDM, Inc., Portland, OR, USA) to ensure the expected amount of data were present. Raw data were then processed using a fully automated, custom MATLAB (Version 2023b; The Math Works Inc., Natick, MA, USA) program. This program is openly available at [https://github.com/jorlandoDPT/Sensors-Paper]. Figure 2 provides a visual representation of the data processing steps. The data were divided into a development and a validation dataset. The development dataset included the first 8 participants, and then the validation dataset included the remaining 2 participants. Raw acceleration components were concatenated for the 8 participants in the development dataset. Figure 2 displays the data processing steps applied to the development dataset. The raw acceleration data were transformed to a world frame of reference using sensor accelerometer, gyroscope, and magnetometer data in a sensor fusion algorithm created and openly shared by APDM [42]. The gravity vector was subtracted from the “up” portion of the world frame data, and then the resultant was calculated to obtain the signal of interest, with acceleration due to gravity removed. To determine appropriate filtering procedures, we then conducted a power spectral density analysis on the resultant acceleration signal and determined the frequency threshold that contained 95% of the signal’s power. We then filtered the acceleration data with a 4th-order lowpass Butterworth filter with a cutoff frequency of 19.9 m/s^2^. The absolute value of the filtered acceleration signal was then downsampled by a factor of 4 (i.e., 128 to 32 Hz) to better correspond to the frame rate of the video data [43].

**Figure 3:**
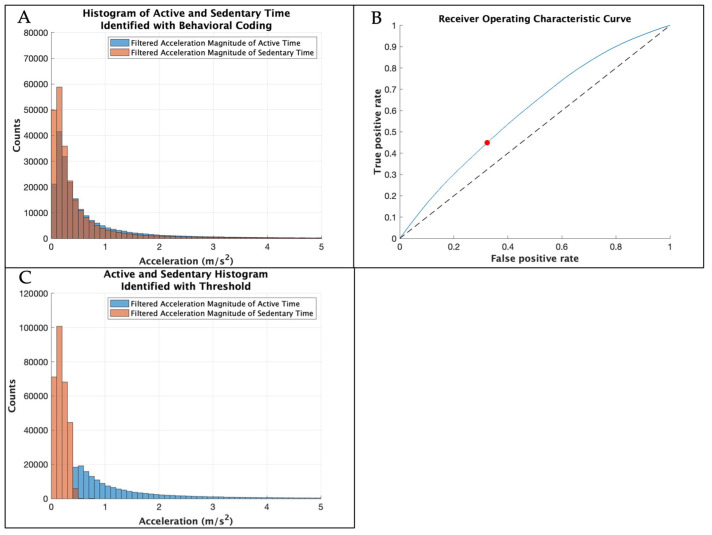
(**A**) Histogram showing the filtered resultant acceleration magnitude of the sensor data during active and sedentary time identified through the gold-standard behavioral coding of the development dataset with the x-axis limit set to 5, excluding higher accelerations with low counts. (**B**) Receiver operating characteristic curve in blue with the optimal threshold in red identified for the development dataset. The threshold was then tested with the validation dataset. (**C**) Histogram showing the filtered resultant acceleration magnitude of the sensor data for the development dataset during active and sedentary time identified by the threshold with the x-axis limit set to 5, excluding higher accelerations with low counts.

Figure 3 displays the steps to determine the threshold to distinguish active from sedentary time from the concatenated 8 participants in the development dataset. A receiver operating characteristic (ROC) curve was plotted for the development dataset using the perfcurve function in MATLAB. The perfcurve function computed the optimal operating point to identify the optimal threshold that maximized true positives and minimized false positives. This threshold is consistent with a visual inspection of the histogram in Figure 3A.

We validated the threshold with the remaining 2 participants (validation dataset). The validation data were processed using the same steps described above. The raw acceleration components were transformed to a world frame of reference, the gravity vector was subtracted from the “up” portion of the world frame data, and then the resultant acceleration magnitude was calculated. The data were filtered using the procedures determined by the development dataset, and then the absolute value of the filtered acceleration signal was downsampled by a factor of 4.

After applying the threshold to each of the participants within the validation dataset, we evaluated the agreement between sedentary and active time identified through the threshold compared to the gold-standard behavioral coding with a tolerance of 0.125 s to correct for any between-frame misalignment between the behavioral coding and sensor data. We calculated the sensitivity (i.e., true positives frames/total positive frames) and specificity (i.e., true negatives frames/total negative frames). A sensitivity and specificity of 0.7–0.79 was considered “acceptable”, and greater than 0.8 was considered “good”.

## 3. Results

The inter-rater agreement was 84.8% between coders. Within the development dataset, 46.7% of the sessions were active time and 53.7% of the sessions were sedentary time, and 0.2% of the data were excluded due to adult handling (which represents time when the sensor was likely moving but perhaps not due to the physical activity of the child), identified through behavioral coding. The optimal threshold for the development dataset was 0.417 m/s^2^, and the area under the curve was from 0.60 (Table 2). The optimal threshold identified a total of 36.5% active time and 63.5% sedentary time.

**Figure 4:**
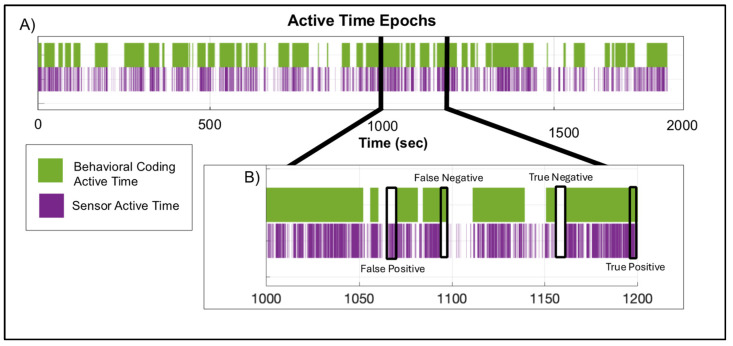
(**A**) Time-series plot displaying the active time epochs identified by the gold-standard behavioral coding (purple) and sensor (green) methodologies for a participant in the validation dataset. (**B**) A subsection of the same time-series displaying examples of true positives [(the sum of the number of frames when the sensor method and the video coding method ± four frames (i.e., 0.125 s) were both classified as active time/total number of frames) × 100], true negatives [(the sum of the number of frames when the sensor method and the video coding method ± four frames (i.e., 0.125 s) were both classified as sedentary time/total number of frames) × 100], false positives [(the sum of the number of frames when the sensor method was classified as active and was not a true positive/total number of frames) × 100], and false negatives [(the sum of the number of frames when the sensor method was classified as sedentary and was not a true negative/total number of frames) × 100].

The percent of true positives and true negatives (i.e., agreement) combined for the validation dataset using the threshold compared to the gold-standard behavioral coding was 92.2% and 89.7%, respectively (Figure 4). The sensitivity and specificity were greater than 85% for both participants (Table 3). Video coding identified 60.0% and 81.0% active time compared to 58.9% and 77.2% active time identified using the threshold for both participants 9 and 10, respectively. Therefore, using video validation with children in the target population, we were able to achieve good agreement with manual behavior coding using an acceleration threshold of 0.417 m/s^2^, above which represented active time and below which represented sedentary time.

## 4. Discussion

This study established the criterion validity of a method of quantifying active and sedentary activity using triaxial accelerometer data from an inertial sensor worn on the lateral thigh for pre-ambulatory children with CP during floor play time. Our approach to distinguish between active and sedentary time suggests potential for good sensitivity and specificity. Importantly, analysis was performed on the raw triaxial accelerometer data, making it applicable to data collected with any inertial sensor as opposed to limited to specific sensors.

In the present study, our primary aim was to distinguish active time; however, previous studies have established sedentary thresholds using activity counts. Oftedal et al. validated cut-points using activity counts to measure sedentary time (i.e., sitting or lying) in non-ambulatory toddlers with CP, and their work may provide a model for continued work validating wearable sensors with young children [23]. However, because their work uses activity counts, there is limited generalizability beyond a specific type of sensor. While measuring the amount of sedentary time is important for health and wellness, identifying active time may be more impactful for early therapeutic interventions.

A known problem with the use of sensors to objectively measure activity with young children is that caregivers may interact with and handle the child through picking up the child, moving the child, or completing usual care, such as diaper changes [44,45,46]. Because we chose to evaluate specific play time sessions, this problem was minimal in our dataset (<1%). We excluded picked-up time from the development dataset and classified this as sedentary time for the validation dataset. Oftedal et al.’s study also identified periods of adult interactions in their validation of cut-points for toddlers with CP using activity counts; however, they excluded time with adult interactions from their analysis [23]. While removing adult interactions does improve the accuracy of sensor-identified activity levels, this does not reflect the naturalistic environment of the child, especially the pre-ambulatory child. Additionally, if research groups plan to capture full-day recordings of child activity, excluding all periods of adult interactions is not feasible as it would be almost impossible to identify all occurrences. We sought to minimize the challenge of adult interactions by focusing on floor play time instead of full-day recordings. Further work examining the differences between full-day recordings and targeted floor play time is needed, and adult interference remains a limitation of sensor-identified activity levels.

While considered the gold standard, behavioral coding is resource intensive and requires the researcher to be present, which can interfere with natural behaviors [19]. Other methods have been employed to evaluate activity in natural environments [47,48]. One such method is ecological momentary assessment (EMA) to measure information about infant positioning at home; however, EMA requires families to use a smart phone to log information or reply to a text message about their infants’ position [49,50]. While this method is considered feasible, these studies still require significant parent engagement, require knowledge of the typical frequency and duration of the occurrence of the behavior being studied, and are open to user interpretation based on the questions or prompts [49]. Similar to EMA, sensors also allow researchers to identify infant behavior in a natural environment and require less effort for families [33].

Another consideration for identifying activity in young children is the location and number of sensors. For the current study, a single sensor placed on the lateral thigh of the child’s dominant leg was chosen to capture transitions and movement consistent with our definition of active time. Many studies involving preschool children have used a hip or low back placement [10,30,51,52,53,54]. Trost et al. compared hip and wrist placement to identify activity in preschoolers and found that either location resulted in an acceptable accuracy of activity recognition with optimal cut-points, with a sensitivity ranging between 88.4 and 89.4 and specificity ranging from 85.1 to 85.8 for recordings at the hip and a sensitivity ranging between 61.3 and 80.2 and specificity ranging from 90.1 to 93.9 for recordings at the wrist [30]. The sensitivity and specificity reported at both locations were similar to the current study. Trost et al. also reported that combining wrist and hip placement had the greatest accuracy; however, using multiple sensors adds expense, processing time, data storage requirements, and additional steps for parents when donning devices [30]. Wrist placement is often selected when evaluating arm use after constraint-induced therapy or detecting infant arm movement [55,56]. In our previous work with preschool children, we found that a few children were interested in the device and attempted to remove it, thus having the device on the wrist may be more accessible than the hip and pose additional challenges within this young population [57].

The accuracy of identifying active and sedentary time is dependent on the definition of active and sedentary time used during behavioral coding. As shown in Supplementary Material S1, our definition of active time focuses on gross movements of the trunk, including movement at the upper or lower extremities that induces trunk movement. We defined sedentary time as any time that was not active, including time with adult interference. In the literature, including studies with children with CP, sedentary time is often indicated by sitting or lying positions [28]. However, sitting is a position where object exploration often occurs, and therefore sitting may be active time if exploratory upper extremity movements also induce trunk movements, such as leaning forward or rotating the trunk to reach objects [6,58]. Clear, consistent descriptions of what constitutes active time are necessary and will enhance the reproducibility of the sensor threshold application in identifying active and sedentary periods during floor play.

## 5. Limitations

This paper has several limitations. The threshold was developed using data from eight participants, and criterion validity was established with two participants, and therefore there is potential for overfitting. The ratio of 80% development and 20% validation data was selected to have enough training data to achieve a threshold that fits the population. The participants in the validation dataset were both GMFCS level I, which may limit the generalizability of the threshold. Importantly, despite their similar general motor ability, these two participants had different amounts of active time, which was reflected in both the behavioral coding and the wearable sensor method. Additionally, while we attempted to provide toys and surfaces to replicate a natural play setting, this analysis did not take place in a natural setting, and therefore the validity of this threshold in a natural setting is not known [28]. Another limitation is the use of a single sensor to identify movement (although this is also a strength in regard to participant burden and resource use). The use of a single sensor could lead to misidentifying active time; to mitigate this limitation, the sensor placement fit our definition of active and sedentary time for this study. Additional limitations include potentially misidentifying active time as sedentary time if the activity involved trunk movement that was below the determined threshold, or misidentifying sedentary time as active time if the child was picked up or moved by an adult in the validation dataset. Importantly, this work established the criterion validity of sensor data from a single thigh sensor during 30 min play intervals for pre-ambulatory children with cerebral palsy; we did not evaluate the validity of this threshold in all circumstances. Future work is needed to assess the validity of this threshold in larger datasets, including children across GMFCS levels, and in natural settings.

## 6. Conclusions

A method of quantifying active and sedentary activity using an inertial sensor for pre-ambulatory children with CP was developed and the criterion validity was established with good sensitivity and specificity. We report the development and validation approach that may serve as a model for validating raw accelerometry data in other populations.

## Figures and Tables

**Table 1:** Participant characteristics.

Participant	Sex	Age (Months)	GMFCS ^^^
1	F	21.9	III
2	M	16.9	V
3	F	15.9	I
4	F	16.0	III
5	F	11.6	I
6	M	9.4	V
7	M	16.3	IV
8	F	16.6	II
9	F	13.8	I
10	M	12.8	I

^^^ GMFCS = Gross Motor Function Classification System [8].

**Table 2:** The optimal threshold and area under the curve for the development dataset and the confusion matrices using the optimal threshold.

Study ID	Optimal Threshold (m/s/s)	Area Under the Curve	Sum of True Positives and True Negatives ^+^	True Positives ^+^ (%)	True Negatives ^+^ (%)	False Positives ^+^ (%)	False Negatives ^+^ (%)
1–8	0.417	0.60	**75.1**	27.9	47.2	6.9	17.9

^+^ Tolerance of 0.125 s; bold font denotes a summary measure.

**Table 3:** Confusion matrices for each participant in the validation dataset using the average threshold.

Study ID	Sum of True Positives and True Negatives ^+^	True Positives ^+^ (%)	True Negatives ^+^ (%)	False Positives ^+^ (%)	False Negatives ^+^ (%)	Sensitivity	Specificity
9	**92.2**	75.2	17.0	2.0	5.8	0.93	0.90
10	**89.7**	53.8	35.9	4.3	6.1	0.90	0.89
Mean	**90.9**	64.5	26.5	3.1	5.9	0.91	0.89

^+^ Tolerance of 0.125 s; bold font denotes a summary measure.

## Data Availability

The data presented in this study are available on request from the corresponding author. The data are not publicly available due to human subject privacy regulations. De-identified data may be shared on request with successful execution of the data use agreement.

## Supplementary Materials

The following supporting information can be downloaded at: https://www.mdpi.com/article/10.3390/s25041261/s1.

## Abbreviations

The following abbreviations are used in this manuscript:

CP	Cerebral palsy
GMFCS	Gross Motor Function Classification System
ROC	Receiver operating characteristic
EMA	Ecological momentary assessment
