# Supplementary material for: Physical Activity in Pre-Ambulatory Children with Cerebral Palsy: An Exploratory Validation Study to Distinguish Active vs. Sedentary Time Using Wearable Sensors
Source: Sensors (Basel). 2025 Feb 19;25(4):1261. doi: 10.3390/s25041261 (PMC11860784; doi:10.3390/s25041261)
Supplement: Supplementary file 1 [file sensors-25-01261-s001.zip › sensors-3435777-supplementary.pdf]

Supplementary Materials S1

Behavioral Coding Scheme

General Guidelines:

- Regardless of posture (standing, tall kneeling), no or minimal movement is defined as sedentary, including small range movements of trunk and lower extremities if child is not directionally moving towards a new position in space (e.g., tapping a foot, wiggling)
- Dynamic movement within a posture OR transitions to different postures (kneeling, 4-point, standing, lying) are "active"
- Label segments of time down to the nearest 1/3 of a second and allow a movement to finish before labeling sedentary.
- Label time as "r" for remove when the child is completely picked up by the therapist

Example scenarios:

- Child is standing, hands resting on foam block, lifts arm (body doesn't change position- just mobility from shoulder) would be "Sedentary", unless arm lifting causes other movement
- Child is lifting up onto tippy toes in standing would be "Active"
- Child is standing and then demonstrates postural sway requiring them to recover their own balance would be "Active"

**Position**	**Active/Movement Description**
Standing	**Head and Trunk motion:** Head and/or trunk movement > 30 degrees in any direction<br>**Leg motion:** marching or stepping, hip or knee flexion consistent with squatting or returning to standing from a squatting position<br>**Arm motion:** Reaching (moving arm at the shoulder) which induces trunk movement (i.e., trunk moves greater than 30 degrees)
Sitting	**Head and Trunk motion:** Head and/or trunk movement > 30 degrees in any direction<br>**Leg motion:** marching or stepping, raising to standing/squatting or lowering from standing/squatting<br>**Arm motion:** Reaching (moving arm at the shoulder) which induces trunk movement (i.e., trunk moves greater than 30 degrees)
Kneeling	**Head and Trunk motion:** Head and/or trunk movement > 30 degrees in any direction<br>**Leg motion:** knee-walking, sliding legs or moving legs in a stepping pattern, transition to another position (short to tall kneeling, ½ kneeling to tall kneeling)<br>**Arm motion:** Reaching (moving arm at the shoulder) which induces trunk movement (i.e., trunk moves greater than 30 degrees)
Quadruped	**Head and Trunk motion:** Head and/or trunk movement > 30 degrees in any direction<br>**Leg motion:** knee-walking, sliding legs or moving legs in a stepping pattern, transition to another position or crawling

	**Arm motion:** Reaching (moving arm at the shoulder) which induces trunk movement (i.e., trunk moves greater than 30 degrees)
Prone	**Head and Trunk motion:** Head and/or trunk movement > 30 degrees in any direction (moving into a prop on elbows or extended arm position<br>**Leg motion:** knee-walking, sliding legs or moving legs in a stepping pattern, transition to another position or crawling<br>**Arm motion:** Reaching (moving arm at the shoulder) which induces trunk movement (i.e., trunk moves greater than 30 degrees)
Supine	**Head and Trunk motion:** Head and/or trunk movement > 30 degrees in any direction, rolling<br>**Leg motion:** lifting legs, kicking or lifting hips in a bridge position<br>**Arm motion:** Reaching (moving arm at the shoulder) which induces trunk movement (i.e., trunk moves greater than 30 degrees)
